# Analysis of Population Structure and Selection Signature in Wadi Sheep Based on Resequencing Data

**DOI:** 10.3390/vetsci13070636

**Published:** 2026-06-30

**Authors:** Zhihua Wang, Te Pi, Yanling Ren, Cuiping Wang, Yishan Li, Feng Li, Shouqing Yan

**Affiliations:** 1 College of Animal Science, Jilin University, Changchun 130062, China; 2 Shandong Binzhou Institute of Animal Husbandry and Veterinary Science, Binzhou 256600, China

**Keywords:** genetic diversity, population structure, selection signature, Wadi sheep, whole-genome sequencing

## Abstract

The Wadi sheep (WAD) is a unique Chinese indigenous breed previously reported to show adaptation to humid and saline–alkali lowlands, disease resistance, high fecundity, and favorable meat quality. However, the recent widespread introduction of commercial sheep breeds has led to a sharp decline in the purebred WAD population, posing serious threats to the preservation of its unique genetic resources. Therefore, there is an urgent need to assess the current population structure and genetic diversity of WAD to support its conservation and sustainable utilization. In this study, we systematically analyzed genomic variation, population structure, and selection signatures using whole-genome resequencing data from 30 WAD sheep and 80 publicly available genomes representing five other breeds obtained from the NCBI database. Population genomic analyses revealed that WAD retains substantial genetic diversity and exhibits a distinct population structure, shaped by its unique breeding history. Selection signature analyses using *F*_ST_, π ratio, and XP-EHH identified 457 candidate genes under positive selection, which are associated with key biological processes including environmental adaptation, immune defense, muscle traits, growth, and reproduction. These findings identify genomic regions potentially related to reported WAD traits and provide a theoretical basis for its conservation, molecular breeding, and sustainable utilization in lowland environments.

## Simple Summary

The Wadi sheep (WAD) is an important local breed in Shandong Province, China. It is well known for its adaptability to humid and saline–alkali lowland environments and has been reported to show disease resistance and year-round reproductive activity. However, the population of purebred WAD has declined in recent decades because of the widespread introduction of other sheep breeds, threatening the long-term preservation of its unique genetic resources. In this study, we used whole-genome data to investigate the genetic characteristics of WAD and compared it with several other breeds. Our results showed that WAD still maintains a relatively high level of genetic diversity and forms a clearly separate genetic group, reflecting its unique breeding history. We also identified many genomic regions shaped by long-term natural and human-driven selection. These regions contain genes potentially related to environmental adaptation, immune defense, muscle traits, growth, and reproduction. These findings suggest possible genomic adaptation of WAD to challenging lowland environments and provide valuable guidance for its conservation and future breeding efforts.

## 1. Introduction

Environmental stressors adversely affect the health and productivity of domestic animals; however, domestication and animal breeding have also made livestock useful models for studying adaptation mechanisms [1]. Sheep (*Ovis aries*), domesticated for more than 10,000 years, have long been essential to agriculture, providing meat, milk, and wool [2]. Under long-term natural selection and selective breeding, a wide range of native sheep breeds have been formed in China, many of which possess distinct genetic characteristics and local adaptive traits [3]. Recent genomic studies on domestic sheep and their wild relatives, along with reviews of livestock selection signatures, demonstrate that genome-wide analyses effectively characterize genetic diversity, adaptive variation, and candidate selection signals in sheep and other livestock [4,5].

The Wadi sheep (WAD), a prominent local breed of Shandong Province, is primarily distributed in the Yellow River Delta of the Lubei Plain. Over time, WAD has developed strong adaptations to the local environment, including tolerance to humid, saline-alkali lowlands, disease resistance, and roughage tolerance [6]. WAD also shows reproductive characteristics such as early sexual maturity and year-round estrus, but it has limitations in meat production, including slow late-stage growth and a relatively low dressing percentage [7]. Therefore, WAD has often been used as the maternal parent in crossbreeding programs [8]. Since the late 20th century, the introduction of commercial breeds such as Dorper and Suffolk has led to a decline in the purebred WAD population. A recent report estimated that the total WAD population in Zhanhua District was approximately 100,600, whereas only approximately 400 purebred individuals were maintained in one WAD conservation farm [9]. This reduction in purebred individuals threatens the retention of WAD’s valuable traits, including environmental adaptability, high fecundity, and disease resistance [10]. Given the increasing risk of losing these traits, it is imperative to characterize the current population structure and genetic architecture of WAD to inform effective conservation and sustainable utilization strategies.

Long-term natural and breeding selection have shaped sheep breeds, generating genomic signatures of selection. Detecting and analyzing these signals has become a key focus in livestock genomic research, including studies on cattle, pigs, chickens, and sheep [11,12,13,14]. Previous studies on WAD have mainly focused on germplasm characterization, crossbreeding improvement, candidate gene analyses, and molecular marker analyses, such as polymorphisms associated with litter size [15]. However, genome-wide analyses of population structure, genetic diversity, and selection signatures in WAD remain limited. In this study, we generated whole-genome sequencing (WGS) data from 30 WAD individuals, integrated public genomic datasets from five other sheep breeds, and identified single nucleotide polymorphisms (SNPs) for population genomic analyses. We aimed to determine whether WAD is genetically distinct from other breeds, whether it retains substantial genetic diversity, and which genomic regions may reflect candidate selection signals potentially related to its reported local adaptation and economically relevant traits. This study provides genomic information for the conservation and sustainable utilization of WAD.

## 2. Materials and Methods

### 2.1. Sample Collection and Sequencing

In this study, blood samples from 30 adult WAD individuals (25 females and 5 males) were collected from a conservation breeding herd at Shandong Jizhi Biotechnology Engineering Co., Ltd. (Binzhou, China). Individuals were selected based on pedigree records to minimize close relatedness. Genomic DNA was extracted from the collected blood samples using the TIANamp Blood DNA Kit (TIANGEN Biotech, Beijing, China). A paired end library was constructed for each individual, and sequencing was performed on the DNBSEQ T7 platform using a 2 × 150 bp paired end sequencing strategy at Novogene Bioinformatics, Beijing, China. Additionally, genomic data for 80 individuals representing five breeds were downloaded from the National Center for Biotechnology Information (NCBI) database, including Dorset sheep (DOR, *n* = 20), Merino sheep (MER, *n* = 20), Tan sheep (TAN, *n* = 10), Small-tailed Han sheep (STH, *n* = 10), and Hu sheep (HUS, *n* = 20).

### 2.2. Reads Mapping and Variant Identification

Raw reads were filtered using fastp (v0.20.1) with default settings [16]. Clean reads were mapped to the Ovis aries reference genome (ARS-UI_Ramb_v3.0) using BWA-MEM (v0.7.13), employing the ‘bwa mem’ algorithm [17]. BAM alignment files were sorted by coordinate using SAMtools (v1.19) [18]. Potential duplicate reads were marked using the MarkDuplicates module in Picard (v1.115) with default parameters [19]. Subsequently, variant calling was performed using the Genome Analysis Toolkit (GATK v4.1.4) [20]. “HaplotypeCaller” was run in GVCF mode for each sample, followed by joint genotyping across all 110 individuals using “GenotypeGVCFs”. SNPs were extracted using “SelectVariants” and filtered using the “Variant Filtration” module of GATK based on the following criteria: ‘QD < 2.0||FS > 60.0||MQ < 40.0||SOR > 3.0||MQRankSum < −12.5||ReadPosRankSum < −8.0’. Genomic SNPs were further filtered using PLINK (v1.9) with the parameters ‘--geno 0.05 --mind 0.1 --maf 0.01’, yielding 32,869,541 high-quality autosomal biallelic variants from 110 individuals [21].

Specifically for the WAD population, genomic data from 30 WAD individuals were filtered using PLINK with the parameters ‘--geno 0.05 --mind 0.1 --maf 0.05’. This Minor Allele Frequency (MAF) threshold was used to maintain a comparable minimum minor allele count with the full dataset, given the smaller sample size of the WAD population. The resulting high-quality SNPs were used for downstream analyses. The allele frequencies of autosomal SNPs in WAD were calculated using VCFtools (v0.1.16) with the ‘--freq’ option, and the functional effects of these SNPs were annotated using SnpEff (v5.1d) with the ARS-UI_Ramb_v3.0 genome annotation file downloaded from NCBI [22,23]. To evaluate the potential functional consequences of the identified SNPs, variant effects were annotated using the Variant Effect Predictor (VEP v115.2) based on the Ensembl ARS UI Ramb v3.0 assembly and its corresponding Ensembl transcript annotation. Functional consequence terms, including missense variant, splice region variant, and start lost, were retained. SIFT scores were generated using the Sorting Intolerant From Tolerant (SIFT v6.2.1) algorithm, with variants scoring ≤ 0.05 considered potentially deleterious [24,25].

### 2.3. Population Structure and Phylogenetic Analysis

The neighbor-joining (NJ) tree was reconstructed using Nei’s genetic distance matrix generated by VCF2Dis (v1.47), and the resulting topology was visualized with SplitsTree (v4.19.2) [26,27,28]. Furthermore, the PLINK software was used to remove SNPs in high linkage disequilibrium (LD) using the parameter ‘--indep-pairwise 50 25 0.2’. The filtered dataset was then used for principal component analysis (PCA), and admixture analysis. PCA was performed using GCTA (v1.92.3) with the ‘--grm’ option to discern genetic relationships among breeds [29]. The first three components were visualized as a three-dimensional PCA plot in R (v4.4.1) using the plotly package (https://plotly.com/), with individuals distinguished by breed-specific colors and point shapes. Population structure was inferred using ADMIXTURE (v1.3). The number of ancestral populations (K) was tested from K = 2 to K = 6, corresponding to the six sheep breeds included in this study. For each K, 20 independent runs were performed using different random seeds to ensure convergence and reduce stochastic variation. Cross-validation (CV) analysis (--cv option) was used to evaluate model fit and determine the optimal K. Graphical representation of the ADMIXTURE results was generated using R [30].

### 2.4. Genetic Diversity Analysis and Linkage Disequilibrium

Genetic diversity within each breed was assessed using the previously filtered high-quality variants. The observed heterozygosity (*H*_O_) and expected heterozygosity (*H*_E_) were estimated using PLINK with the ‘--hardy’ option. Furthermore, the nucleotide diversity (π) was determined using VCFtools with the parameters ‘--window-pi 50,000 --window-pi-step 25,000’. Runs of homozygosity (ROH) were identified using PLINK based on the following parameters: a minimum of 50 SNPs per ROH (--homozyg-snp 50), a sliding window size of 50 SNPs (--homozyg-window-snp 50), allowance of up to five missing SNPs and three heterozygous SNPs per window (--homozyg-window-missing 5 and --homozyg-window-het 3), a minimum ROH length of 300 kb (--homozyg-kb 300), a minimum SNP density of one SNP per 50 kb (--homozyg-density 50), a maximum gap between consecutive SNPs of 1000 kb (--homozyg-gap 1000), and a window threshold of 0.05 (--homozyg-window-threshold 0.05) [31]. The ROH-based inbreeding coefficient (*F*_ROH_) was calculated as the proportion of the autosomal genome covered by ROH segments with the total autosomal genome length in this study. The number and total length of ROHs per individual were calculated for each breed and classified into three size categories (<1 Mb, 1–3 Mb, and ≥3 Mb) to assess the distribution patterns of short and long ROHs across breeds [32]. Breed-level values are presented as mean ± standard deviation (SD) across individuals within each breed. Linkage disequilibrium (LD) decay was evaluated by calculating the correlation coefficient (r^2^) of pairwise SNPs using PopLDdecay (v3.42) with default parameters [33].

### 2.5. Analysis and Identification of Selective Sweeps

To detect potential genomic regions related to selection in WAD, genome-wide selection signatures were identified using three complementary methods: fixation index (*F*_ST_), nucleotide diversity ratio (π ratio), and cross-population extended haplotype homozygosity (XP-EHH). HUS and STH were combined as the reference group for all three selection scans because they had suitable sample sizes and relatively comparable regional and production backgrounds to WAD. The combined reference group included 20 HUS and 10 STH individuals, resulting in the same sample size as WAD (*n* = 30). *F*_ST_ between WAD and the combined reference group was calculated using VCFtools with the parameters “--fst-window-size 50,000 --fst-window-step 25,000”. Nucleotide diversity (π) was calculated separately for WAD and the combined reference group using VCFtools with the parameters “--window-pi 50,000 --window-pi-step 25,000” [34]. For the selection scan based on nucleotide diversity, the π ratio was calculated as −ln(π_WAD_/π_REF_), where π_WAD_ and π_REF_ represent the nucleotide diversity of WAD and the reference population, respectively. Higher π ratio values indicate a greater reduction in nucleotide diversity in WAD relative to the reference population. Haplotype phasing and genotype imputation were conducted using BEAGLE (v5.4), and the resulting phased genotypes were used to compute XP-EHH scores with Selscan (v1.2). XP-EHH values were calculated and normalized using the ‘-norm’ function [35]. Subsequently, candidate windows were defined as the empirical top 5% of windows with the highest scores for each statistic, following previous selection signature studies [36]. To improve the reliability of candidate region detection, the top ranked windows identified by *F*_ST_, π ratio, and XP-EHH were intersected using the intersect function in BEDtools (v2.30.0) [37]. Genomic regions shared by all three methods were defined as putative selected regions. Genes located within these shared regions were annotated as candidate genes using SnpEff to evaluate their potential functional effects. In addition, haplotype patterns in candidate regions were visualized using ComplexHeatmap (v2.25.2), and functional effects of variants were assessed using VEP and SIFT scores [38]. To explore the biological significance of candidate genes, Gene ontology (GO) and Kyoto Encyclopedia of Genes and Genomes (KEGG) pathway enrichment analyses were performed using DAVID (https://davidbioinformatics.nih.gov). Raw *p*-values and Benjamini–Hochberg adjusted *p*-values were reported, and terms with adjusted *p*-values < 0.05 were considered significantly enriched [39,40]. Additionally, QTL data were downloaded from the Sheep QTLdb (Release 57, released on 26 August 2025; accessed on 1 December 2025) to identify QTL intervals overlapping with the candidate selected regions [41]. QTL overlap was assessed by positional intersection between the candidate selected regions and QTL intervals using BEDtools.

## 3. Results

### 3.1. Sequencing and SNPs Calling

We performed WGS on 30 WAD individuals, generating 1106.27 Gb of raw data. After quality filtering, 1100.74 Gb of clean data were obtained. The sequencing depth ranged from 10.93× to 34.72× across the 30 individuals, with an average depth of 13.0×. The sequencing data achieved a 99.8% mapping rate, with Q20 > 99% and Q30 > 96% (Supplementary Table S1). Additionally, 80 publicly available WGS datasets representing five sheep breeds were obtained from the NCBI SRA and processed together with the WAD individuals. The breed information, SRA Run accession numbers, and BioProject accession numbers of these public datasets are listed in Supplementary Table S2.

Following quality control of the SNP dataset using PLINK, 22,741,411 biallelic SNPs were identified in WAD. The SNP dataset showed a transition/transversion ratio (Ts/Tv) of 2.524 and a genome-wide SNP density averaging 9.301 SNPs/kb. The distribution of SNP density varied among chromosomes, with values ranging from 8.365 SNPs/kb (chromosome 11) to 10.160 SNPs/kb (chromosome 25). Functional annotation of the identified SNPs revealed that the majority were located in intergenic (49.848%) and intronic (36.753%) regions, while a mere 0.684% were found to be situated within exonic regions. (Supplementary Table S3). Within exonic regions, 103,319 SNPs (0.454%) were annotated as synonymous variants, 50,913 (0.224%) as missense variants, and 895 (0.004%) as stop-gained mutations. The SNP annotation pattern was similar to that reported in a previous WGS study of Chinese domestic sheep, supporting the reliability of our variant dataset [42]. SIFT prediction identified 3033 deleterious variants (SIFT ≤ 0.05), of which 1045 had a SIFT score of 0, indicating the strongest predicted deleterious effect (Supplementary Table S4).

### 3.2. Population Structure and Admixture Analysis

**Figure 1:**
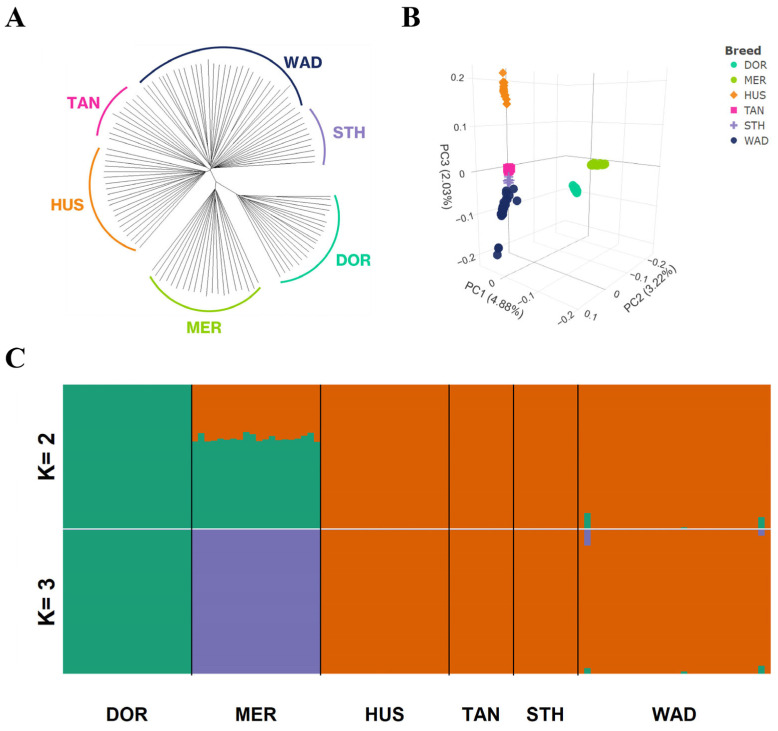
Population structure and relationships among six sheep breeds (110 individuals in total). (**A**) Neighbor-joining tree of the relationships among breeds. (**B**) Principal component analysis. (**C**) Model-based clustering of sheep breeds using ADMIXTURE with K = 2 to 3. In panel (**C**), different colors represent different inferred ancestral components. Abbreviations: DOR: Dorset sheep; MER: Merino sheep; HUS: Hu sheep; TAN: TAN sheep; STH: Small-tailed Han sheep; WAD: Wadi sheep.

The NJ tree grouped WAD with the Chinese indigenous breeds HUS, TAN, and STH, whereas the two exotic breeds, DOR and MER, formed distinct branches (Figure 1A). Furthermore, after LD pruning, 3,809,189 autosomal SNPs were retained for downstream analyses. PCA based on LD-pruned SNPs showed breed level differentiation among the six breeds (Figure 1B). The first principal component (PC1), which explained 4.88% of the total genetic variation, separated the exotic breeds (DOR and MER) from the Chinese indigenous breeds. The second component (PC2, 3.22%) showed a similar differentiation pattern between the exotic and Chinese indigenous breeds. The third component (PC3, 2.03%) further differentiated HUS from the remaining Chinese indigenous breeds, while TAN, STH, and WAD were positioned near each other. The structure analysis ranged from K = 2 to 6 with 20 independent runs per K. Cross-validation analysis identified K = 2 as the model with the lowest CV error (Supplementary Figure S1). At K = 2, DOR formed a distinct genetic cluster, whereas MER showed an admixed ancestry pattern, sharing components with both exotic and Chinese indigenous breeds. At K = 3, MER was further separated into a more distinct ancestry component, while DOR remained clearly differentiated. The Chinese indigenous breeds (HUS, TAN, STH, and WAD) remained closely grouped (Figure 1C). These results were consistent with the NJ tree and PCA analyses, which showed clear separation between exotic and Chinese indigenous breeds and close genetic relationships among Chinese indigenous sheep.

### 3.3. Genetic Diversity and Linkage Disequilibrium Patterns

**Figure 2:**
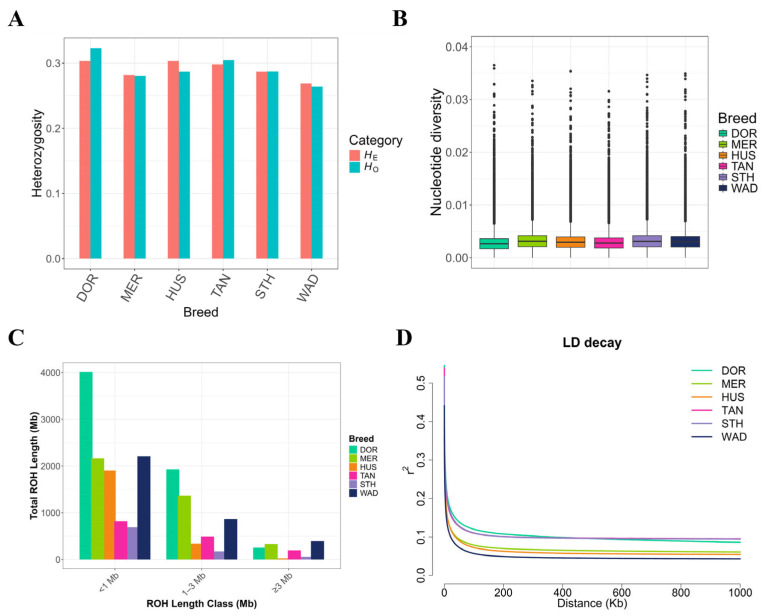
Summary statistics for genomic variation in 110 individuals from 6 breeds. (**A**) *H*_O_ and *H*_E_ of each breed. (**B**) Box plots of π for each breed. (**C**) Distribution of total ROH length across different ROH length classes among the six sheep breeds. ROH segments were classified into <1 Mb, 1–3 Mb, and ≥3 Mb. (**D**) The decay of LD on sheep autosomes was estimated for each breed.

Genome-wide diversity of WAD was assessed by calculating *H*_O_, *H*_E_, π, and *F*_ROH_ from 32,869,541 autosomal biallelic SNPs, in comparison with five reference breeds, including two exotic breeds (DOR and MER) and three indigenous Chinese breeds (TAN, STH, and HUS). WAD exhibited a relatively lower *H*_O_ (0.2641 ± 0.0143) and *H*_E_ (0.2686 ± 0.0002) compared to the other five reference populations (Figure 2A). However, its nucleotide diversity (π = 0.0032 ± 0.00011) was higher than that observed in HUS (0.0031 ± 0.00007), TAN (0.0028 ± 0.00013), and DOR (0.0029 ± 0.00014, Figure 2B). Across all breeds, most ROH segments fell within the <1 Mb category. DOR exhibited the highest cumulative ROH length in both the <1 Mb and 1–3 Mb categories compared with the other breeds. Within indigenous sheep populations, WAD showed relatively higher cumulative ROH length in the <1 Mb category than HUS, TAN and STH. In the long ROH category (≥3 Mb), WAD exhibited the highest cumulative ROH length among all breeds. Overall, the distribution of ROH length varied among breeds, indicating distinct patterns of genomic homozygosity across indigenous and imported sheep populations (Figure 2C). Regarding inbreeding, WAD showed a moderate *F*_ROH_ value (0.0468 ± 0.0461), which was higher than STH (0.0373 ± 0.0087) and HUS (0.0460 ± 0.0048) but substantially lower than DOR (0.1254 ± 0.0321) and MER (0.0782 ± 0.0333). As a locally developed breed with a relatively limited population size, WAD has nonetheless retained a substantial level of genetic variation. In addition, LD decay patterns varied among breeds. WAD showed the fastest LD decay and the lowest LD levels as marker distance increased, suggesting relatively low genome-wide linkage disequilibrium. This pattern may reflect the combined effects of recombination history, effective population size, selection, and demographic history. In contrast, DOR showed the slowest LD decay and the highest LD levels, which may be associated with reduced genetic diversity and stronger selective breeding in this introduced breed (Figure 2D).

### 3.4. Genome-Wide Scanning for Selection Signatures

**Figure 3:**
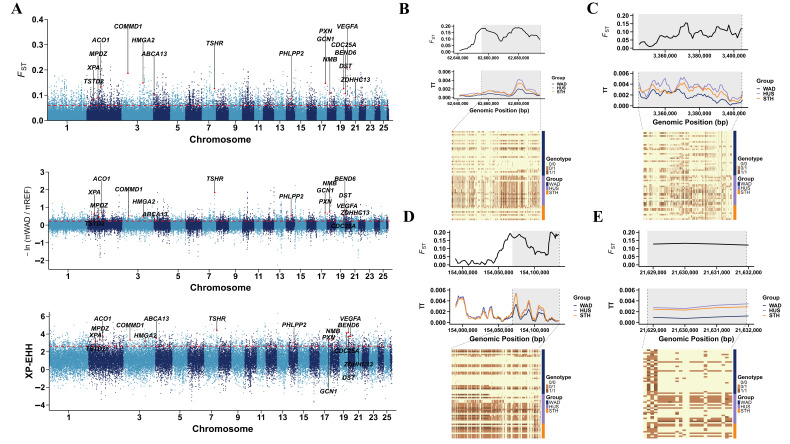
Genome-wide selection scans and visualization of representative candidate regions in WAD. (**A**) Analysis of the signatures of positive selection in the genome of WAD using *F*_ST_, π ratio, and XP-EHH. The dashed lines indicate the top 5% threshold for each statistic, and candidate genes located near selected regions are labeled. (**B**–**E**) Visualization of selection signals and local genotype patterns in representative regions harboring *GCN1*, *BEND6*, *HMGA2*, and *NMB*. For each region, the upper two plots show *F*_ST_ and π, respectively, and the lower heatmaps show genotype patterns among WAD, HUS, and STH. The shaded regions indicate candidate windows. In the genotype heatmaps, homozygous reference genotypes (0/0), heterozygous genotypes (0/1), and homozygous alternate genotypes (1/1) are shown in light yellow, brown, and dark brown, respectively. The side color bar indicates population groups, including WAD, HUS, and STH.

In the selection signal analyses, *F*_ST_, π ratio, and XP-EHH methods were employed to identify candidate regions and genes. The top 5% of regions were designated as candidate selection signatures. As a result, *F*_ST_, π ratio, and XP-EHH identified 3337, 3809, and 2419 genes, respectively (Figure 3A). Integration of the top 5% of regions from all three methods revealed 1865 candidate regions, within which 457 overlapping genes were detected (Figure 3B). The genomic coordinates and corresponding selection statistics of candidate regions identified by each method are provided in Supplementary Tables S6–S8, and the shared candidate regions and associated genes are listed in Supplementary Table S9.

Among the 457 candidate genes located in regions detected by all three selection scan methods, 17 genes were prioritized for focused discussion according to published evidence and previously reported functional annotations related to the main traits considered in this study, including environmental adaptation, immune response, growth, muscle traits, and reproduction (Table 1). To provide more precise gene trait information, Table 1 summarizes both the broad trait category and the functional description of each prioritized gene. Genes involved in environmental adaptation included *ABCA13*, *GCN1*, *TSTD2*, *VEGFA*, *XPA*, and *ZDHHC13*, which are implicated in stress response, angiogenesis, and skin barrier function. Immune-related genes such as *BEND6*, *COMMD1*, *PHLPP2* and *ACO1* were also identified, potentially reflecting selective pressure from pathogenic environments. In addition, *MPDZ* has previously been associated with footrot resistance in sheep [43]. For muscle traits, *DST* and *PXN* were identified, likely contributing to muscle structure and meat quality. *HMGA2*, a gene implicated in growth and metabolic processes, was associated with selection on growth traits. Reproduction-related genes included *CDC25A*, *NMB*, and *TSHR*, which have been linked to litter size and labor onset in previous studies. To further characterize candidate regions under selection, four representative genes located within WAD selective sweep regions (*GCN1*, *BEND6*, *HMGA2*, and *NMB*) were selected for local visualization of selection signals and genotype patterns (Figure 3B–E).

GO and KEGG enrichment analyses of the 457 candidate genes identified 38 GO terms and 17 KEGG pathways, with raw *p* values and Benjamini–Hochberg adjusted *p* values provided in Supplementary Table S10. After Benjamini–Hochberg correction, two KEGG pathways remained significant. The first was “microRNAs in cancer” (*p* = 1.94 × 10^−4^; Benjamini = 0.0285), which was considered a broad KEGG annotation category and was not further interpreted in relation to WAD adaptation. The second was “retinol metabolism” (*p* = 2.38 × 10^−4^; Benjamini = 0.0285), a pathway with potential relevance to physiological regulation in livestock. In addition, several selected regions overlapped with functional QTLs, including fecal egg count and pleurisy QTLs related to immune response; body weight and tail fat deposition QTLs associated with growth; offspring number and teat number QTLs linked to reproduction; milk fat yield QTLs related to lactation; and fleece yield, staple and length QTLs associated with wool production. These overlaps suggest that selection in WAD may be shaped by disease pressure, productivity requirements, and the challenges posed by saline–alkaline environments (Supplementary Table S11).

## 4. Discussion

Recent advances in high-throughput sequencing technologies have greatly enhanced the resolution and scope of livestock genomic research [60]. Compared with traditional approaches such as microsatellites or SNP chips, WGS provides genome-wide variant information, enabling more comprehensive analyses of population structure, genetic diversity, and selection signatures [61]. Although WAD is recognized for its valuable traits, genome-wide studies are still lacking, which limits our understanding of its genetic basis and hampers targeted conservation and breeding efforts. Given WAD’s unique environmental adaptation and high fecundity, WGS serves as a crucial tool to elucidate its genetic architecture and support future conservation and utilization.

Population structure analyses based on the NJ tree, PCA, and ADMIXTURE consistently showed that WAD clustered with Chinese indigenous breeds, particularly TAN and STH, and was clearly separated from the exotic breeds DOR and MER. PCA further supported this pattern, with WAD individuals clustering closely with indigenous breeds along the first three principal components. The proportion of variance explained by PC1–PC3 was relatively low, which is commonly observed in within-species livestock population analyses where genetic variation is distributed across multiple dimensions of the genome rather than being captured by a few principal axes [34,42]. The concordant clustering patterns observed across the three analytical approaches indicate a stable and consistent population stratification among the studied breeds. Together with its geographic and breeding background, these results suggest that WAD represents a distinct local genetic resource adapted to lowland environments.

In this study, genome-wide diversity analysis showed that WAD exhibits moderate to high levels of genetic variation. Specifically, *H*_O_, *H*_E_, and π in WAD were comparable to or slightly higher than those in other Chinese native breeds, and slightly lower than those in MER. These results indicate that, despite its relatively small population size and geographically restricted distribution, WAD has retained substantial allelic diversity. Moreover, the ROH landscape provides insights into the demographic history and population structure of each breed [62]. In the present study, DOR exhibited the highest cumulative ROH length in both the short (<1 Mb) and medium (1–3 Mb) categories among all breeds. Within indigenous sheep populations, WAD showed relatively higher cumulative ROH length in the <1 Mb class compared with TAN and STH, consistent with previous reports in Yabuyi, Karakul, and Wadi sheep, where elevated ROH levels were observed across multiple length categories, reflecting breed-specific patterns of genomic homozygosity rather than a single demographic process [63]. In the long ROH category (≥3 Mb), WAD displayed the highest cumulative ROH length among all breeds, indicating a relatively higher proportion of long homozygous segments. Such long ROH segments are generally considered to reflect recent genomic homozygosity influenced by breeding history and population structure [64]. The genomic inbreeding coefficient *F*_ROH_ of WAD was slightly higher than those of the other Chinese native breeds but lower than those of the exotic breeds DOR and MER, indicating a moderate level of homozygosity. Furthermore, WAD exhibited the most rapid LD decay and the lowest LD extent among all breeds analyzed. These findings underscore the genetic uniqueness of WAD and highlight its potential as a valuable local resource for future conservation and breeding initiatives.

Positive natural selection enhances adaptation by increasing the frequency of beneficial genetic variants, thereby improving survival in specific environments [65]. Although TAN is genetically closer to WAD in population structure analyses, it was not used as a reference population due to its distinct northwestern dryland origin and long-term pelt-oriented selection history. Instead, HUS and STH were selected as reference groups given their more comparable regional and production backgrounds, thereby reducing potential confounding from divergent ecological and selection histories in scans for economically relevant traits in WAD. Using three complementary methods, we identified 457 overlapping candidate genes located in candidate selected regions of WAD. These results suggest that environmental pressures and long-term artificial selection may have contributed to the genomic differentiation of WAD, particularly in relation to adaptation to saline–alkali lowland environments.

Environmental adaptation emerged as one of the major candidate selection signals in WAD, possibly reflecting long-term pressure from humid and saline–alkali conditions. Several genes may be involved in stress tolerance, epidermal protection, oxidative balance, and vascular adaptation. *VEGFA* has been reported to promote placental and peripheral angiogenesis, which may support vascular remodeling under environmental stress [5,47]. *GCN1* regulates the integrated stress response by modulating translation during amino acid deprivation and oxidative imbalance. In this study, the *GCN1* region showed genotype differentiation between WAD and the reference breeds, suggesting that this region may represent a candidate stress response-related locus in WAD [45]. *ZDHHC13* is involved in protein palmitoylation and skin barrier development, which may be relevant to epithelial protection under humid and variable environments [49]. *ABCA13* encodes an ABC transporter and has been reported as a candidate gene in sheep adaptation studies, suggesting a possible role in membrane transport-related physiological responses [44]. In addition, *XPA* facilitates nucleotide excision repair of UV- and oxidative DNA damage [48]. *TSTD2* belongs to the sulfurtransferase-related gene family and may be involved in sulfur and redox metabolism, which is relevant to oxidative stress regulation [46]. Collectively, these genes represent candidate loci potentially involved in epithelial and cellular stress responses in WAD, although their precise roles in environmental adaptation require further functional validation.

Moreover, several candidate genes were related to immune regulation and epithelial barrier function. *COMMD1* regulates NF-κB signaling and transcriptional control of inflammatory pathways [51]. *PHLPP2* downregulates macrophage-driven inflammation through Akt dephosphorylation [52]. *ACO1* functions in iron homeostasis and T cell immune modulation [53]. *BEND6* has been reported to inhibit IRF3 activation and suppress antiviral signaling [50]. In this study, the *BEND6* region showed genotype differentiation between WAD and the reference breeds, suggesting that this region may represent an immune signaling-related candidate locus in WAD. *MPDZ* has been identified as a candidate gene for footrot resistance or susceptibility in sheep, possibly through its role in tight junction integrity and interdigital skin barrier function, which may be relevant under humid environmental conditions [43].

In addition to genes related to environmental adaptation and immune response, several selected regions contained genes associated with muscle traits, growth, and reproduction. *DST* and *PXN* are involved in cytoskeletal organization and myotome morphogenesis, respectively, supporting their relevance as muscle-related candidate genes [54,55]. For growth-associated selection, *HMGA2* has been consistently linked to core sheep growth traits including body height and body weight [56]. The *HMGA2* region also showed visible genotype differentiation between WAD and the closely related reference breeds. Reproductive genes under selection included *CDC25A*, *NMB*, and *TSHR*, among which the *NMB* region showed genotype differentiation between WAD and the reference breeds. Previous studies have linked *CDC25A* and *TSHR* variants with litter size and *NMB* with labor onset, suggesting that these genes may represent candidate reproductive loci in WAD [57,58,59].

These findings provide important insights into the genomic basis of the reported adaptive and productive traits of WAD and highlight its distinct genomic background compared with exotic and other indigenous breeds. However, this study has several limitations, including the lack of phenotypic association analysis and functional validation, the limited WAD sample size, and potential biases associated with public reference datasets. Therefore, the candidate genes and selection signals identified here require further validation in future studies. Despite these limitations, the results provide valuable molecular resources for future conservation breeding and sustainable genetic improvement of sheep adapted to humid lowland environments, while emphasizing the importance of protecting the genetic integrity of this irreplaceable local resource.

## 5. Conclusions

In conclusion, this study provides a comprehensive overview of genomic variation in WAD using whole-genome resequencing. We showed that WAD retains substantial genetic diversity and exhibits a distinct population structure, despite facing threats of genetic dilution. A total of 457 candidate genes located in selected regions were identified and were potentially related to environmental adaptation, immune response, muscle traits, growth, and reproduction. These findings improve our understanding of the genetic basis underlying the unique traits of WAD and provide valuable genomic resources for the conservation and sustainable genetic improvement of WAD, with particular attention to maintaining the genetic integrity of purebred populations in humid and saline-alkali lowland environments.

## Figures and Tables

**Table 1:** Candidate genes in selected regions potentially associated with adaptive and economically relevant traits in WAD.

Chromosome	Position (bp)	Candidate Genes	Traits	Functional Description
4	7,991,622–8,385,199	*ABCA13*	Environmental adaptation	ABC membrane transport and substrate transport [44]
17	62,640,026–62,694,475	*GCN1*	Environmental adaptation	Integrated stress response and translation regulation [45]
2	50,023,657–50,051,932	*TSTD2*	Environmental adaptation	Sulfur metabolism and redox regulation [46]
20	17,279,780–17,295,199	*VEGFA*	Environmental adaptation	Angiogenesis and hypoxia-related vascular response [47]
2	49,855,538–49,997,011	*XPA*	Environmental adaptation	Nucleotide excision repair and DNA damage response [48]
21	23,308,566–23,363,530	*ZDHHC13*	Environmental adaptation	Protein palmitoylation and skin barrier development [49]
20	3,344,345–3,405,012	*BEND6*	Immune response	Innate antiviral immune regulation [50]
3	46,085,935–46,274,005	*COMMD1*	Immune response	Copper metabolism and inflammatory regulation [51]
2	81,610,406–82,278,670	*MPDZ*	Immune response	Tight junction integrity linked to footrot resistance [43]
14	38,877,694–38,941,305	*PHLPP2*	Immune response	NF κB inflammatory signaling regulation [52]
2	101,546,097–101,611,247	*ACO1*	Immune response	Iron metabolism and T cell immune modulation [53]
20	3,405,362–3,929,524	*DST*	Muscle integrity	Cytoskeletal organization and muscle integrity [54]
17	62,702,917–62,745,901	*PXN*	Muscle development	Focal adhesion and myotome morphogenesis [55]
3	153,989,269–154,134,744	*HMGA2*	Growth	Body size and growth regulation [56]
19	51,698,291–51,721,756	*CDC25A*	Reproduction	Cell cycle regulation related to fecundity [57]
18	21,628,718–21,632,001	*NMB*	Reproduction	Uterine contraction and labor onset [58]
7	90,438,630–90,610,132	*TSHR*	Reproduction	Regulating folliculogenesis and sheep litter size [59]

## Data Availability

The data presented in this study will be openly available. The sequencing reads generated in this study have been deposited in the NCBI Sequence Read Archive (SRA) under the BioProject accession number PRJNA1280530.

## Supplementary Materials

The following supporting information can be downloaded at https://www.mdpi.com/article/10.3390/vetsci13070636/s1. Supplementary Table S1: Summary of sequencing data; Supplementary Figure S1: Cross-validation error for different K values (K = 2–6) in the population structure analysis; Supplementary Table S2: Summary of 80 sheep sample information; Supplementary Table S3: Distribution of SNPs identified in WAD annotated by SnpEff; Supplementary Table S4: List of deleterious missense SNPs in WAD based on SIFT predictions; Supplementary Table S5: Genetic diversity of the 6 sheep breeds used in this study; Supplementary Table S6: A summary of genes from *F*_ST_ in WAD; Supplementary Table S7: A summary of genes from π ratio in WAD; Supplementary Table S8: A summary of genes from XP-EHH in WAD; Supplementary Table S9: A summary of genes overlapped by *F*_ST_, π ratio, and XP-EHH methods in common regions; Supplementary Table S10: GO and KEGG enrichment analysis of WAD candidate genes by three methods; Supplementary Table S11: QTLs overlapped with candidate selected regions.
